# Comprehensive Analysis of the Prognostic Value and Immune Infiltration of Butyrophilin Subfamily 2/3 (BTN2/3) Members in Pan-Glioma

**DOI:** 10.3389/fonc.2022.816760

**Published:** 2022-08-10

**Authors:** Dong He, Zhen Qin, Zihao Liu, Xiaoshuai Ji, Jiajia Gao, Hua Guo, Fan Yang, Haitao Fan, Yanbang Wei, Zixiao Wang, Qian Liu, Qi Pang

**Affiliations:** ^1^ Department of Neurosurgery, Shandong Provincial Hospital Affiliated to Shandong First Medical University, Jinan, China; ^2^ Department of Neurosurgery, Shandong Provincial Hospital, Cheeloo College of Medicine, Shandong University, Jinan, China; ^3^ Department of Histology and Embryology, Cheeloo College of Medicine, School of Basic Medical Sciences Shandong University, Jinan, China; ^4^ Department of Clinical Laboratory, Affiliated Hospital of Shandong University of Traditional Chinese Medicine, Jinan, China; ^5^ Department of Neurosurgery, Shandong Provincial Qianfoshan Hospital, Cheeloo College of Medicine, Shandong University, Jinan, China

**Keywords:** BTN/BTNL, BTN2/3, pan-glioma, immune infiltration, biomarkers

## Abstract

The BTN2/3 subfamilies are overexpressed in many cancers, including pan-glioma (low- and high-grade gliomas). However, the expression and prognosis of BTN2/3 subfamilies and tumor-infiltrating lymphocytes in pan-glioma remain unknown. In the present study, we systematically explored and validated the expression and prognostic value of BTN2/3 subfamily members in pan-glioma [The Cancer Genome Atlas–glioblastoma and low-grade glioma (TCGA-GBMLGG) merge cohort] using multiple public databases. We used clinical specimens for high-throughput verification and cell lines for qRT-PCR verification, which confirmed the expression profiles of BTN2/3 subfamilies. In addition, the function of the BTN2/3 subfamily members and the correlations between BTN2/3 subfamily expression and pan-glioma immune infiltration levels were investigated. We found that BTN2/3 subfamily members were rarely mutated. BTN2/3 subfamilies were overexpressed in pan-glioma; high expression of BTN2/3 subfamily members was correlated with poor prognosis. In addition, BTN2/3 subfamilies might positively regulate proliferation, and the overexpression of BTN2/3 subfamilies influenced cell cycle, differentiation, and glioma stemness. In terms of immune infiltrating levels, BTN2/3 subfamily expression was positively associated with CD4+ T-cell, B-cell, neutrophil, macrophage, and dendritic cell infiltrating levels. These findings suggest that BTN2/3 subfamily expression is correlated with prognosis and immune infiltration levels in glioma. Therefore, the BTN2/3 subfamilies can be used as biomarkers for pan-glioma and prognostic biomarkers for determining the prognosis and immune infiltration levels in pan-glioma.

## Introduction

Glioma is the most prevalent primary malignant brain tumor with the characteristics of aggressive growth and invasiveness to surrounding tissues. It accounts for nearly 80% of primary malignant intracranial tumors and 30% of central nervous system tumors (1). Due to its aggressive and invasive nature, completely resecting the glioma tissue in surgery is impractical (2). Moreover, its resistance to radio/chemotherapy can be easily induced after long-term treatment, which leads to the vulnerability of relapse (3).

By regulating the immune system, immunotherapy exhibits superior antitumor efficacy with minimal side effects and has been widely used in a variety of cancers. However, limited progress has been made in the immunotherapy of glioma. Glioma possesses a highly immunosuppressive tumor microenvironment, owing to the presence of a few and/or exhausted tumor-infiltrating lymphocytes and a lack of specific and immunogenic tumor antigens (4). Therefore, it is important to understand the hub genes involved in the immune characteristics of glioma and to explore more effective therapeutic targets.

In our previous study, it was found that FAM111A was involved in regulating immune function in low-grade glioma (LGG) (5). On this basis, we further found that members of the BTN (butyrophilin)/BTNL (butyrophilin-like) subfamilies were highly correlated with the expression status of FAM111A, which caught our attention. BTN/BTNL molecules have been emerging as novel regulators of immune responses in humans (6–8). For example, BTN3A3 is the LSECtin receptor involved in the stemness regulation of breast cancer in tumor-associated macrophages (TAMs) (9). BTN2A1 plays a pivotal role in phosphoantigen reactivity in γδ T cells and can directly bind to the germline-encoded regions of the Vγ9Vδ2 TCR for phosphoantigen sensing (10). In addition, the development of several γδ IEL compartments is based on the epithelial expression of encoding genes, in which butyrophilin-like [Btnl (mouse) or BTNL (human)] members of the B7 superfamily of T-cell costimulators are involved (11). However, in the field of glioma immunotherapy, the relationship between abnormal expression of BTN/BTNL and immune characteristics remains unknown.

In the present study, we first analyzed the expression and prognostic value of BTN/BTNL in pan-glioma using Level 3 HTseq-FPKM format RNA-seq data and clinical data in The Cancer Genome Atlas–glioblastoma and LGG (TCGA-GBMLGG) dataset. According to the characteristics of differential expression, the research scope was narrowed to BTN2/3 subfamily members (BTN2A1, BTN2A1, BTN3A1, BTN3A2, and BTN3A3). The correlation between BTN2/3 subfamily expression and immune inhibitors was investigated, as well as its association with infiltrating immune cells, by utilizing TISIDB and TIMER. To the best of our knowledge, this is the first comprehensive study of associations between the expression of BTN2/3 subfamily genes and their immunological characteristics, molecular signature, and clinical performance in pan-glioma. Our results may help to optimize immunotherapy for LGG and high-grade glioma.

## Results

### Expression of the BTN/BTNL and BTN2/3 Subfamilies in Pan-Glioma

**Figure 1:**
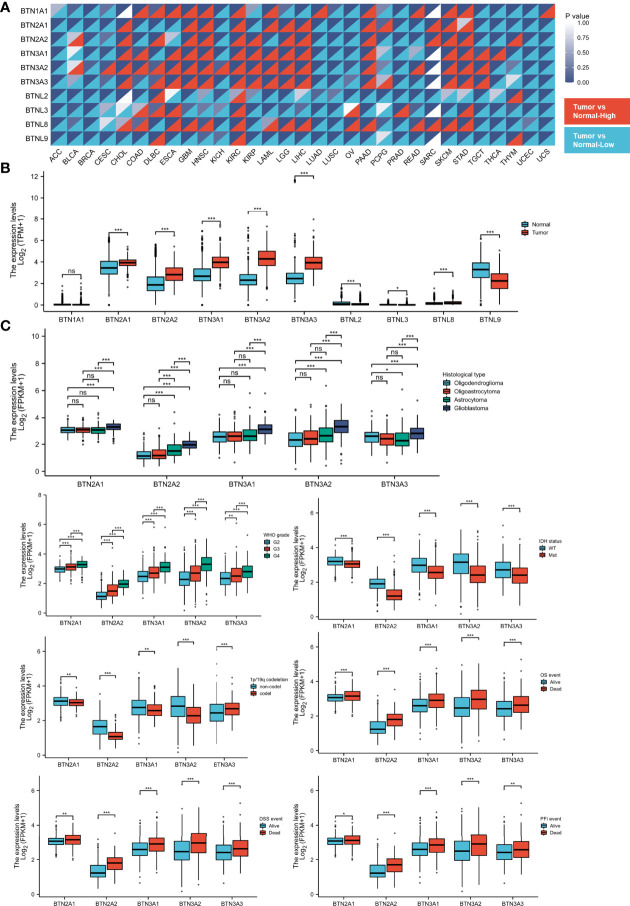
Expression of BTN/BTNL and BTN2/3 in pan-cancer and pan-glioma. **(A)** BTN/BTNL expression levels in different tumor types from TCGA database were determined by R software. Each cell in the heatmap displays the p-value at the upper right and the expression status at the lower right. The high expression in the tumor corresponds to red, and the low expression corresponds to blue. **(B)** BTN/BTNL expression levels in TCGA-GBMLGG merge cohort. **(C)** Correlation between BTN2/3 subfamily expression levels and clinicopathological characteristics (histological type, WHO grade and IDH status, and survival events). BTN, butyrophilin; BTNL, butyrophilin-like; TCGA, The Cancer Genome Atlas; GBMLGG, glioblastoma and low-grade glioma. *P < 0.05; **P < 0.01; ***P < 0.001; “ns” stands for “no significance”.

To identify the differential BTN/BTNL expression pattern between tumor and normal tissue in various types of cancers, the BTN/BTNL mRNA levels were analyzed using Gene Expression Profiling Interactive Analysis (GEPIA). The results showed that the BTN/BTNL expression levels were diversified across cancers, among which the most typical high expression levels in tumors are kidney renal clear cell carcinoma (KIRC), lymphoid neoplasm diffuse large B-cell lymphoma (DLBC), skin cutaneous melanoma (SKCM), and stomach adenocarcinoma (STAD). In adrenocortical carcinoma (ACC), breast invasive carcinoma (BRCA), uterine corpus endometrial carcinoma (UCEC), and uterine carcinoma (UCS) datasets, BTN/BTNL showed a low expression profile in tumor tissues (**Figures 1A** and **S1**). Based on the BTN/BTNL expression intensity, the expression levels of BTN1A1, BTNL2, BTNL3, and BTNL8 in most types of tumors and normal tissues were relatively low (**Figures 1A** and **S1**). Similar results were found in the GBMLGG merge cohort. The BTN2/3 subfamily members showed high expression in tumors and low expression in normal tissues. BTNL9 had a higher basal expression level, and its expression level in normal tissues was higher than that in tumor tissues. Because the basal expression levels of BTN1A1, BTNL2, BTNL3, and BTNL8 were relatively low, in further analysis, we only focused on the expression of BTN2/3 subfamilies in pan-gliomas (**Figure 1B**).

The results of correlation analysis between clinicopathological characteristics and expression showed that the expression level of BTN2/3 subfamilies had a positive correlation with the pathological type and the WHO grades of glioma (**Figure 1C**). IDH wild-type glioma tends to have higher BTN2/3 subfamily expression levels, and IDH wild-type glioma is generally considered to have a poor prognosis. The result of the 1p/19q codeletion was not exactly the same as that of the IDH wild-type/mutant type (**Figure 1C**). BTN3A3 expression was higher in patients with the 1p/19q codeletion. Except for BTN3A3, the expression of other BTN2/3 subfamilies was the opposite. The data on the correlation between overall survival (OS)/disease-specific survival (DSS)/progression-free interval (PFI) and BTN2/3 subfamily expression supported the oncogenic results of BTN2/3 in IDH wild-type glioma. In addition, the expression levels of BTN2/3 subfamilies in deceased patients were higher than those in living patients (**Figure 1C**).

### Genomic Alterations of BTN2/3 Subfamilies and Gene-Gene/Protein-Protein Interaction Network

**Figure 2:**
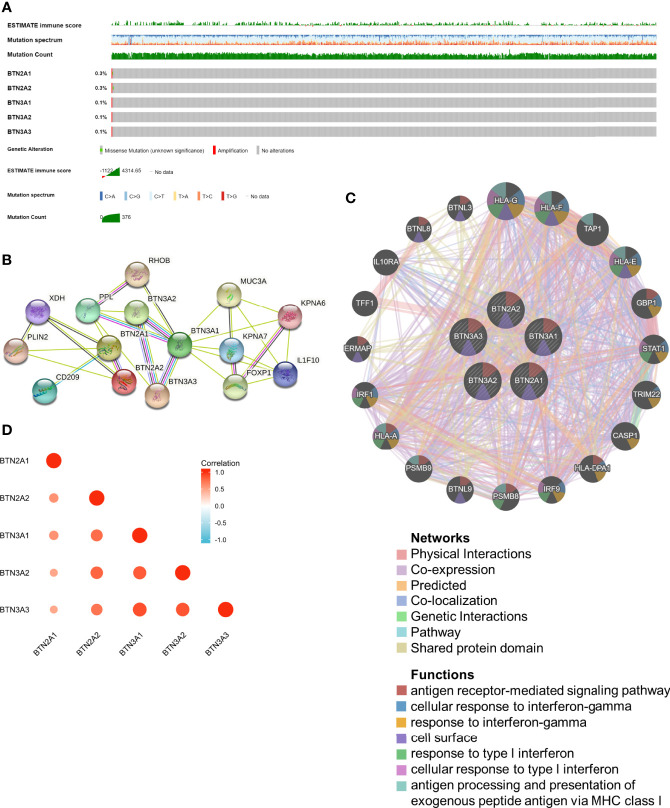
Genomic alterations of BTN2/3 subfamily members and gene–gene and protein–protein interaction networks of genes of the TBC1D3 family. **(A)** The OncoPrint schematic provides an overview of genomic alterations of the BTN2/3 subfamilies in TCGA-GBMLGG merge cohort. **(B)** A network diagram of interactions between proteins encoded by genes of the BTN2/3 subfamilies, drawn using STRING. **(C)** The gene network associated with the BTN2/3 subfamilies, drawn by using GeneMANIA. **(D)** Correlation analysis of BTN2/3 subfamily members. TCGA-GBMLGG, The Cancer Genome Atlas–glioblastoma and low-grade glioma.

cBioPortal was utilized to determine the types and frequencies of BTN2/3 subfamily alterations in TCGA-GBMLGG samples. The results showed that BTN2/3 subfamily members were rarely mutated and were highly conserved (**Figure 2A**).

The gene–gene and protein–protein interaction networks, which were generated by GeneMANIA and STRING, showed the top 20 potential target genes and 10 potential target proteins interacting with the BTN2/3 subfamilies, respectively (**Figures 2B, C**). The functions of potential target genes revealed in GeneMANIA were consistent with previous studies, mainly related to antigen processing and type I interferon. Then, we used “correlation analysis” by GEPIA for five members of the BTN2/3 subfamilies, which showed that associations among members of BTN2/3 subfamilies were positively correlated (**Figure 2D**).

### Prognostic Potential of the BTN2/3 Subfamilies in Pan-Glioma

**Figure 3:**
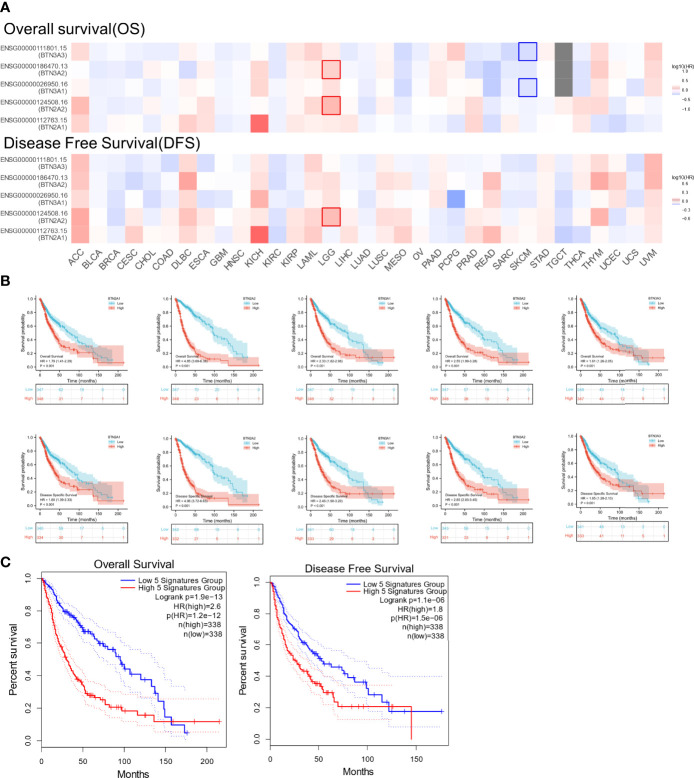
The prognostic value of BTN2/3 subfamily members in pan-glioma. **(A)** Survival map of BTN2/3 subfamily members across 33 cancer types from TCGA. **(B)** Survival curves of BTN2/3 subfamily members were analyzed by R software. Top, overall survival (OS); bottom, disease-specific survival (DSS). **(C)** Survival curves of BTN2/3 subfamily 5 gene signatures were analyzed by R software. TCGA, The Cancer Genome Atlas.

We next investigated whether BTN2/3 subfamily expression was associated with pan-glioma prognosis. The impact of BTN2/3 subfamily expression on survival rates was evaluated using GEPIA. The pan-cancer survival map of the hazardous ratio of BTN2/3 subfamilies was plotted. BTN2/3 subfamilies, as risk factors, were more prominent in the LGG dataset (**Figure 3A**). In addition, higher expression of BTN2/3 subfamilies was associated with shorter survival. OS and DSS analysis indicated that patients with a high expression of the BTN2/3 subfamilies had poor prognoses (**Figure 3B**). Considering the five members of BTN2/3 as the overall genetic signature, the conclusion of the survival analysis was the same as the above (**Figure 3C**).

To further confirm the contribution of BTN2/3 subfamilies to the clinical characteristics of pan-glioma, we investigated the association between BTN2/3 subfamily expression and clinicopathological characteristics using the Product-Limit method (Kaplan–Meier method). As shown in **Table 1**, high BTN2/3 expression was correlated with OS among sexes (except BTN3A3 in women), WHO grade II (BTN2A2), III (BTN2A2, BTN3A1, and BTN3A2), and IDH-Mut (BTN2A2 and BTN3A2) in glioma. To explore whether BTN2/3 subfamily expression was an independent predictor of OS/DSS/PFI in pan-glioma, we performed univariate and multivariate Cox regression analyses. First, in the univariate Cox regression analysis, factors including age, IDH status, and BTN2/3 subfamilies were all independent risk factors for OS/DSS/PFI (**Tables 2**–**4**). In the multivariate Cox regression analysis, in addition to age and IDH status, BTN2A2 and BTN3A1 were risk factors for OS, and BTN2A2, BTN3A1, and BTN3A3 were risk factors for DSS and PFI (**Tables 2**–**4**).

**Table 1:** Correlation of BTN2/3 subfamily mRNA expression and clinical prognosis in GBMLGG with different clinicopathological factors.

		BTN2A1			BTN2A2			BTN3A1			BTN3A2			BTN3A3		
	Total (N)	HR	CI	p	HR	CI	p	HR	CI	p	HR	CI	p	HR	CI	p
Sex																
Female	298	1.51	1.03–2.21	**0.028**	5.04	3.42–7.43	**0.000**	2.2	1.50–3.24	**0.000**	2.25	1.54–3.29	**0.000**	1.25	0.86–1.82	0.237
Male	398	1.96	1.44–2.67	**0.000**	4.11	2.99–5.63	**0.000**	2.35	1.72–3.21	**0.000**	2.67	1.96–3.65	**0.000**	1.81	1.33–2.47	**0.000**
WHO grade																
G1	–	–	–	–	–	–	–	–	–	–	–	–	–	–	–	–
G2	224	0.7	0.36–1.36	0.3	1.95	1.01–3.80	**0.048**	0.59	0.30–1.14	0.114	1.12	0.57–2.17	0.744	0.49	0.25–0.95	0.034
G3	243	0.87	0.56–1.34	0.519	2.13	1.37–3.29	**0.001**	1.74	1.12–2.70	**0.011**	1.75	1.13–2.70	**0.011**	1.28	0.83–1.98	0.265
G4	168	1	0.71–1.39	0.979	0.94	0.67–1.32	0.729	0.74	0.53–1.04	0.075	0.75	0.54–1.05	0.095	0.82	0.58–1.14	0.231
IDH status																
IDH-WT	246	1.2	0.89–1.61	0.214	1.1	0.82–1.48	0.506	1.17	0.87–1.57	0.299	1	0.75–1.35	0.984	1.05	0.78–1.41	0.753
IDH-Mut	440	1.42	0.93–2.16	0.095	2.18	1.43–3.30	**0.000**	1.43	0.94–2.16	0.094	1.68	1.11–2.56	**0.013**	1.24	0.82–1.88	0.309

GBMLGG, glioblastoma and low-grade glioma; HR, hazard ratio.

Bold values indicate statistical significance.

**Table 2:** Univariate and multivariate analyses of the correlation of BTN2/3 subfamily mRNA expression with OS among GBMLGG patients.

Characteristics	Total (N)	Univariate analysis		Multivariate analysis	
		Hazard ratio (95% CI)	p-Value	Hazard ratio (95% CI)	p-Value
Age	695				
≤60	552	Reference			
>60	143	4.668 (3.598–6.056)	**<0.001**	1.931 (1.452–2.568)	**<0.001**
Sex	695				
Female	297	Reference			
Male	398	1.262 (0.988–1.610)	0.062	1.334 (1.025–1.736)	**0.032**
IDH status	685				
WT	246	Reference			
Mut	439	0.117 (0.090–0.152)	**<0.001**	0.197 (0.140–0.278)	**<0.001**
BTN2A1	695				
High	348	Reference			
Low	347	0.557 (0.437–0.711)	**<0.001**	1.039 (0.798–1.354)	0.776
BTN2A2	695				
High	348	Reference			
Low	347	0.206 (0.157–0.271)	**<0.001**	0.570 (0.389–0.835)	**0.004**
BTN3A1	695				
High	348	Reference			
Low	347	0.430 (0.336–0.550)	**<0.001**	0.642 (0.454–0.907)	**0.012**
BTN3A2	695				
High	348	Reference			
Low	347	0.392 (0.305–0.503)	**<0.001**	0.946 (0.663–1.350)	0.760
BTN3A3	695				
High	347	Reference			
Low	348	0.622 (0.489–0.791)	**<0.001**	1.382 (0.985–1.938)	0.061

OS, overall survival; GBMLGG, glioblastoma and low-grade glioma.

Bold values indicate statistical significance.

**Table 3:** Univariate and multivariate analyses of the correlation of BTN2/3 subfamily mRNA expression with DSS among GBMLGG patients.

Characteristics	Total (N)	Univariate analysis		Multivariate analysis	
		Hazard ratio (95% CI)	p-Value	Hazard ratio (95% CI)	p-Value
Age	674				
≤60	541	Reference			
>60	133	4.500 (3.409–5.940)	**<0.001**	1.824 (1.346–2.470)	**<0.001**
Sex	674				
Female	289	Reference			
Male	385	1.248 (0.965–1.614)	0.092	1.269 (0.958–1.680)	0.097
IDH status	664				
WT	232	Reference			
Mut	432	0.110 (0.083–0.146)	**<0.001**	0.180 (0.125–0.258)	**<0.001**
BTN2A1	674				
High	334	Reference			
Low	340	0.555 (0.429–0.717)	**<0.001**	1.032 (0.781–1.365)	0.824
BTN2A2	674				
High	332	Reference			
Low	342	0.201 (0.151–0.269)	**<0.001**	0.600 (0.400–0.899)	**0.013**
BTN3A1	674				
High	333	Reference			
Low	341	0.406 (0.312–0.528)	**<0.001**	0.581 (0.402–0.838)	**0.004**
BTN3A2	674				
High	331	Reference			
Low	343	0.378 (0.290–0.492)	**<0.001**	0.941 (0.646–1.371)	0.751
BTN3A3	674				
High	333	Reference			
Low	341	0.607 (0.470–0.782)	**<0.001**	1.471 (1.025–2.111)	**0.036**

DSS, disease-specific survival; GBMLGG, glioblastoma and low-grade glioma.

Bold values indicate statistical significance.

**Table 4:** Univariate and multivariate analyses of the correlation of BTN2/3 subfamily mRNA expression with PFI among GBMLGG patients.

Characteristics	Total (N)	Univariate analysis		Multivariate analysis	
		Hazard ratio (95% CI)	p-Value	Hazard ratio (95% CI)	p-Value
Age	695				
≤60	552	Reference			
>60	143	2.873 (2.268–3.640)	**<0.001**	1.276 (0.985–1.652)	0.065
Sex	695				
Female	297	Reference			
Male	398	1.083 (0.875–1.342)	0.463		
IDH status	685				
WT	246	Reference			
Mut	439	0.151 (0.119–0.191)	**<0.001**	0.203 (0.151–0.273)	**<0.001**
BTN2A1	695				
High	348	Reference			
Low	347	0.717 (0.579–0.887)	**0.002**	1.149 (0.912–1.449)	0.239
BTN2A2	695				
High	348	Reference			
Low	347	0.295 (0.234–0.371)	**<0.001**	0.644 (0.467–0.889)	**0.007**
BTN3A1	695				
High	348	Reference			
Low	347	0.508 (0.410–0.631)	**<0.001**	0.626 (0.458–0.856)	**0.003**
BTN3A2	695				
High	348	Reference			
Low	347	0.509 (0.410–0.632)	**<0.001**	1.100 (0.817–1.481)	0.529
BTN3A3	695				
High	347	Reference			
Low	348	0.692 (0.560–0.856)	**<0.001**	1.363 (1.017–1.826)	**0.038**

PFI, progression-free interval; GBMLGG, glioblastoma and low-grade glioma.

Bold values indicate statistical significance.

### Functions of the BTN2/3 Subfamilies in Pan-Glioma

**Figure 4:**
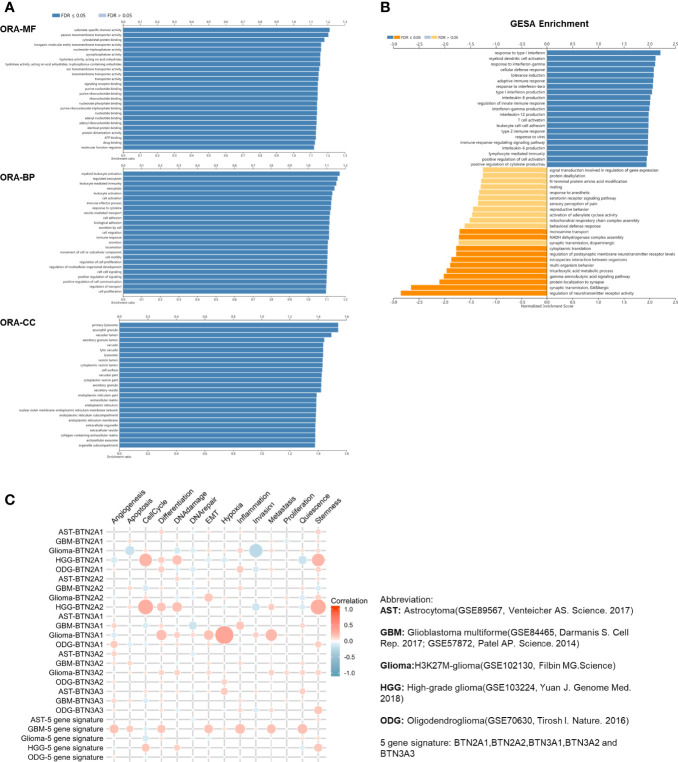
Function of the BTN2/3 subfamilies in pan-glioma. **(A)** The ORA-based biological process (BP), molecular function (MF), and cellular component (CC) of BTN2/3 subfamilies were analyzed by LinkedOmics. **(B)** Gene set enrichment analysis (GSEA) of subfamilies was performed by LinkedOmics. **(C)** Single-cell analysis by CancerSEA indicated that BTN2/3 subfamilies were primarily involved in regulating the cell cycle, differentiation, and stemness. The abbreviations and references of the single-cell datasets are shown in the figure.

To explore the functions of BTN2/3 subfamilies in pan-glioma, we performed overrepresentation enrichment analysis (ORA) by the LinkedOmics online database, which revealed the biological process (BP), molecular function (MF), and cellular component (CC) of BTN2/3 family members (**Figure 4A**). It is worth noting that the principal biological function that the BTN2/3 subfamilies participate in is myeloid leukocyte activation, which is closely related to the immune response. Gene set enrichment analysis (GSEA) helped to reveal the gene pathways involved in the upregulation or downregulation of the BTN2/3 subfamilies. The results showed that the top three upregulated genes were in response to type I interferon, myeloid dendritic cell (DC) activation, and interferon-gamma (**Figure 4B**).

To investigate the function of BTN2/3 subfamilies in pan-glioma, we performed a single-cell analysis using CancerSEA. The results demonstrated that BTN2/3 subfamilies may influence the cell cycle, differentiation, and stemness of pan-glioma (**Figure 4C**).

### The Association Between BTN2/3 Subfamily Expression and Immunosuppression

**Figure 5:**
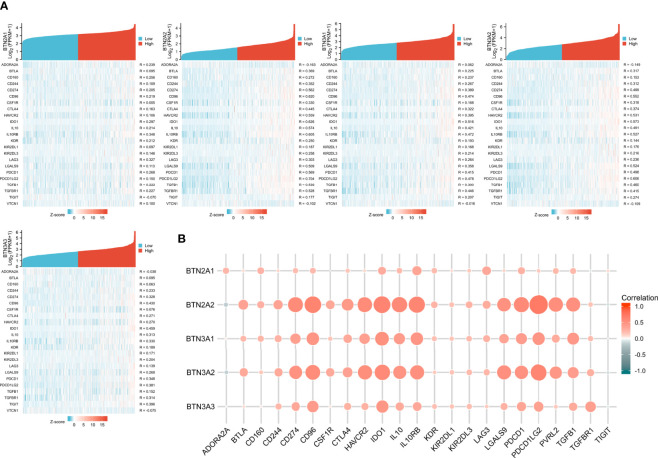
Correlations of BTN2/3 subfamily expression and immune inhibitors in pan-glioma. **(A)** Single-gene correlation heatmap of BTN2/3 subfamilies and the TISIDB immune inhibitory dataset. **(B)** Overview heatmap of BTN2/3 subfamily members and the TISIDB immune inhibitory dataset.

The function and role of novel immune checkpoint inhibitors (ICIs) have attracted attention. Consequently, we assessed whether BTN2/3 subfamily expression was associated with ICIs. The database TISIDB was selected to investigate the association between BTN2/3 subfamily expression and potential immune inhibitory effects. We obtained the correlation data of ICIs and BTN2/3 expression in the TISIDB database and drew a heatmap (**Figure 5A**). The BTN2A1, BTN2A2, BTN3A1, BTN3A2, and BTN3A3 were the five members of the BTN2/3 subfamilies that were associated with CD274, CD96, IDO1, IL10, 1L10RB, PDCD1, PDCD1LG2, and TFGB1 (Spearman’s correlation coefficient, R > 0.2, absolute value). The results of BTN2A2, BTN3A1, and BTN3A2 showed that these three members are related to the expression of various types of novel ICIs and have greater correlation coefficients (**Figure 5B**).

### The Association Between BTN2/3 Subfamily Expression and Immune Infiltration

We applied the Level 3 HTseq-FPKM format RNA-seq data and clinical data in TCGA-GBMLGG dataset and used the gene set variation analysis (GSVA) package of R software and the built-in single-sample GSEA (ssGSEA) algorithm for immune infiltration analysis. The abbreviations and corresponding names of the 24 immune cells were as follows: activated DCs (aDCs); B cells; CD8 T cells; cytotoxic cells; DCs; eosinophils; immature DCs (iDCs); macrophages; mast cells; neutrophils; NK CD56 bright cells; NK CD56dim cells; NK cells; plasmacytoid DCs (pDCs); T cells; T helper cells; T central memory (Tcm); T effector memory (Tem); T follicular helper (Tfh); T gamma delta (Tgd); Th1 cells; Th17 cells; Th2 cells; and Treg.

**Figure 6:**
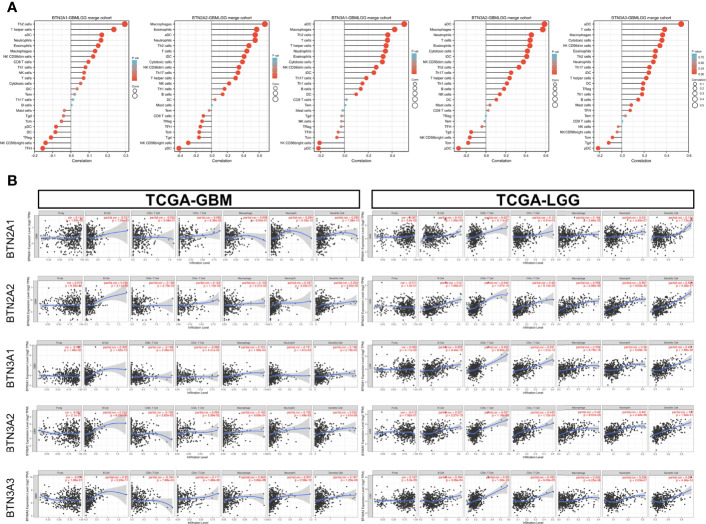
Correlation of BTN2/3 subfamily expression with immune infiltration levels in pan-glioma. **(A)** Level 3 HTseq-FPKM format RNA-seq data and clinical data in TCGA-GBMLGG dataset analyzed by GSVA–ssGSEA immune infiltration analysis. The abbreviations and corresponding names of the 24 immune cells are as follows: aDC, activated DC; B cells; CD8 T cells; cytotoxic cells; DC; eosinophils; iDC, immature DC; macrophages; mast cells; neutrophils; NK CD56bright cells; NK CD56dim cells; NK cells; pDC, plasmacytoid DC; T cells; T helper cells; Tcm, T central memory; Tem, T effector memory; Tfh, T follicular helper; Tgd, T gamma delta; Th1 cells; Th17 cells; Th2 cells; Treg. **(B)** Correlation between BTN2/3 subfamily expression and tumor-infiltrating lymphocytes in pan-glioma by TIMER. TCGA-GBMLGG, The Cancer Genome Atlas–glioblastoma and low-grade glioma; GSVA, gene set variation analysis; ssGSEA, single-sample gene set enrichment analysis.

Our results showed significant correlations, as follows: BTN2A1 expression with most of the marker sets of Th2 cells, T helper cells, and aDCs; BTN2A2 expression with most of the marker sets of macrophages, eosinophils, and aDCs; BTN3A1 expression with most of the marker sets of aDCs, macrophages, and Th2 cells; BTN3A2 expression with most of the marker sets of aDCs, macrophages, and neutrophils; and BTN3A3 expression with most of the marker sets of aDCs, T cells, and macrophages (**Figure 6A**).

**Figure 7:**
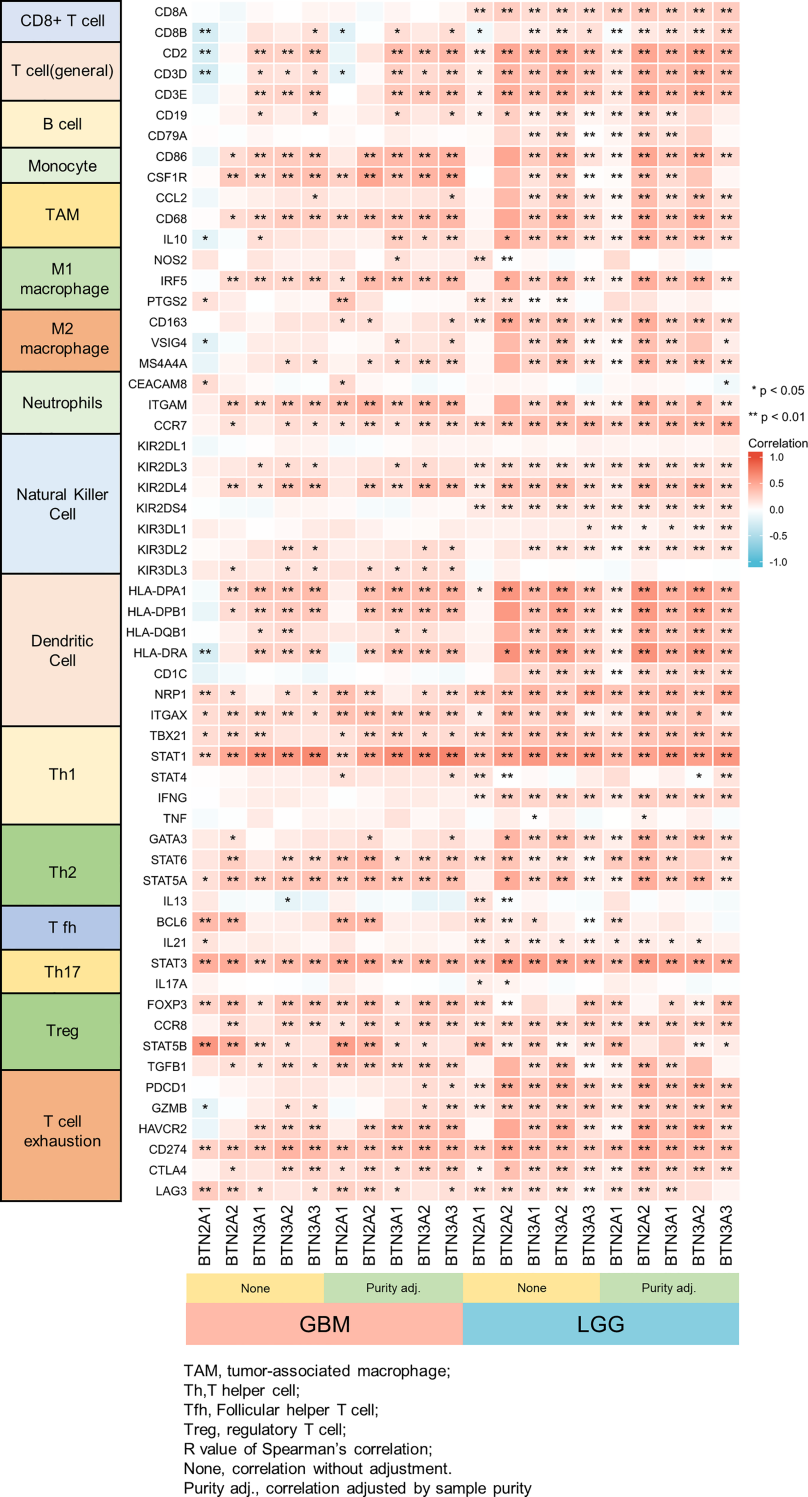
Correlation heatmap between BTN2/3 subfamilies and markers of immune cells in pan-glioma by TIMER. *P < 0.05; **P < 0.01.

We further performed TIMER analysis to investigate the relationship between BTN2/3 subfamily expression and tumor-infiltrating lymphocytes in pan-glioma. BTN2/3 subfamily expression was positively associated with CD4+ T cell, neutrophil, macrophage, and DC infiltration levels (**Figure 6B**). In addition, the infiltration levels of immune cells in the LGG dataset were more significant than those in the GBM dataset. To further explore the relationship between immune cell infiltration levels and BTN2/3 subfamily expression in the GBMLGG dataset, we used TIMER to investigate the correlations between BTN2/3 subfamily expression and various immune infiltration-associated markers. Our results showed that there was a significant correlation between most of the marker sets of Th1, Th2, Treg, and T-cell exhaustion and BTN2/3 subfamily expression (**Figure 7**). Especially for T-cell exhaustion, the results were consistent with the ssGSEA results.

**Figure 8:**
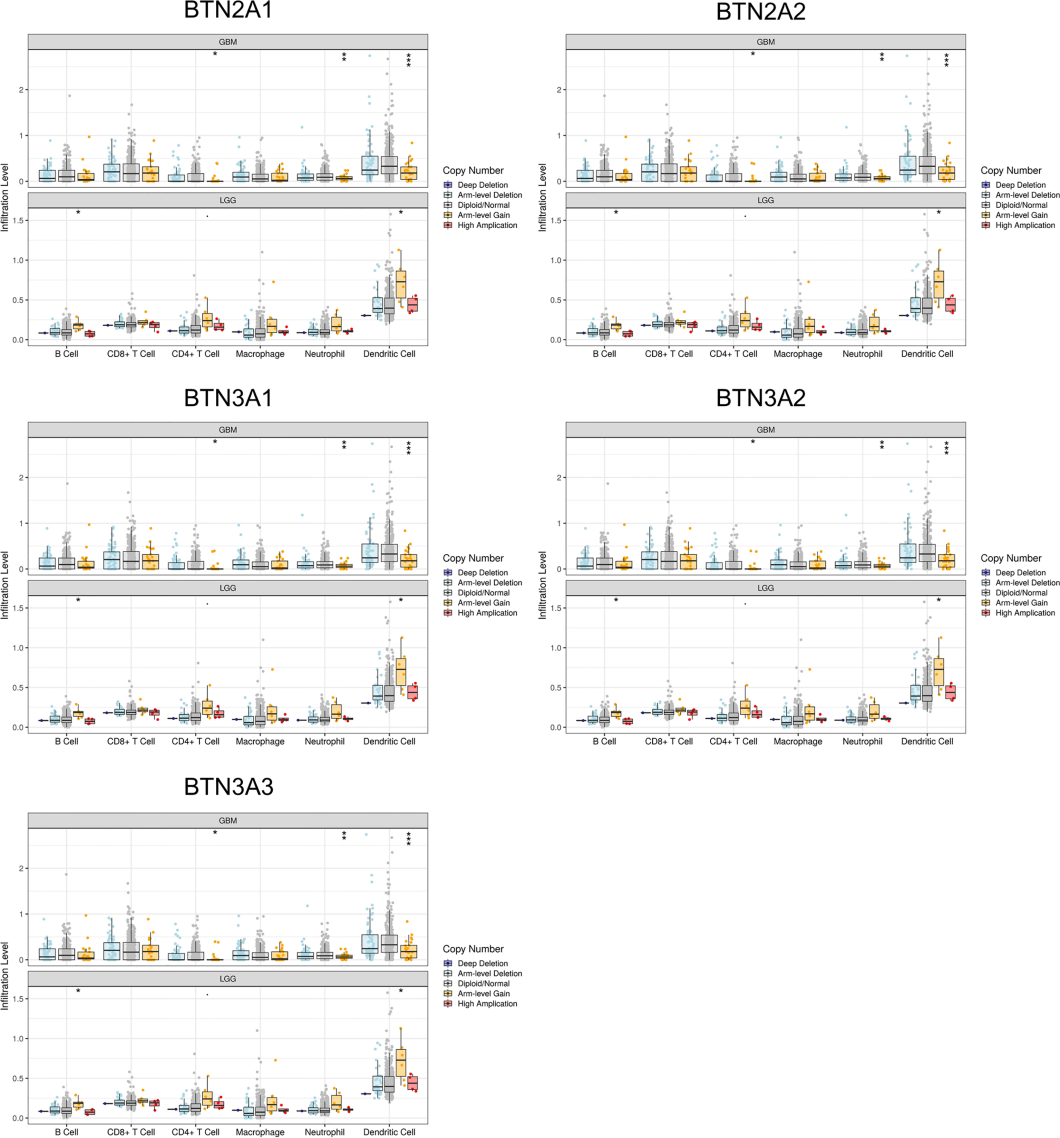
Correlation of tumor-infiltrating levels in GBM/LGG and different somatic copy number alterations in the BTN2/3 subfamily members. The arm-level deletion of BTN2/3 subfamily members was significantly associated with immune cell infiltration levels in GBM, and the arm-level gain of BTN2/3 subfamily members was significantly associated with immune cell infiltration levels in LGG. GBM, glioblastoma; LGG, low-grade glioma. *P < 0.05; **P < 0.01; ***P < 0.001.

The somatic copy number alteration (SCNA) module showed that the arm-level deletion of BTN2/3 subfamily members was significantly associated with immune cell infiltration levels in GBM. In addition, the arm-level gain of BTN2/3 subfamily members was significantly associated with immune cell infiltration levels in LGG (**Figure 8**).

### Verification of BTN2/3 Subfamily Expression Profiles and Immune Infiltration

**Figure 9:**
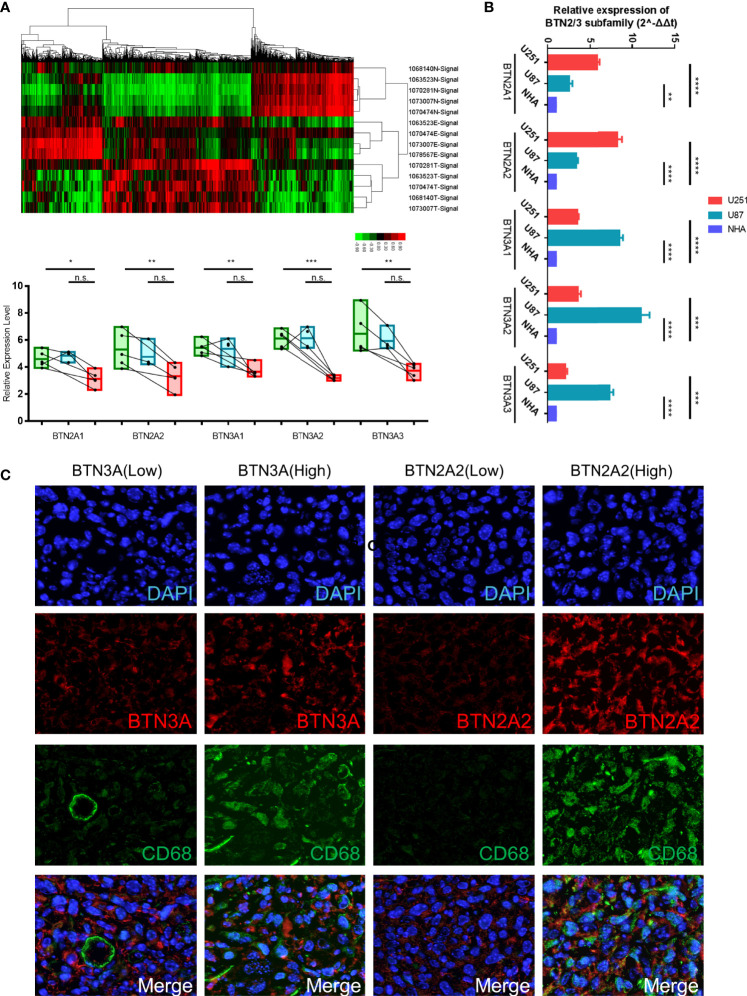
Verification of BTN2/3 subfamily expression profiles and immune infiltration. **(A)** The differential expression clusters of PCGs in the glioma specimens and paracancerous tissues are shown. The suffix E-Signal represents edge tissues, the suffix T-Signal represents tumor tissues, and the suffix N-Signal represents normal tissues. The heatmap indicates the upregulated and downregulated PCGs. The expression levels of BTN2/3 subfamilies in the different tissues are displayed below. **(B)** The differential expression levels of BTN2/3 in the normal human astrocyte (NHA) cell line and GBM cell lines (U87 and U251). The results are presented as the mean ± SD from three independent experiments. **(C)** Results of immunohistochemical fluorescence staining. According to the qRT-PCR results of BTN2A2 and BTN3A, the glioblastoma clinical samples were divided into high-/low-expression groups, and immunohistochemical fluorescence staining was performed. Green signal, CD68, a panmacrophage biomarker; red signal, BTN2A2 or BTN3A, as presented in the figure; blue signal, DAPI. PCGs, protein-coding genes. *P < 0.05; **P < 0.01; ***P < 0.001; ****P < 0.0001; ns, no significance.

To verify the above conclusions, we identified the expression of protein-coding genes (PCGs) in glioma, precancerous, and normal brain samples by using the Affymetrix GeneChip Human Transcriptome Array 2.0 (**Figure 9A**, **Table S1**), and we performed real-time PCR in two GBM cell lines (U87 and U251) and the normal human astrocyte (NHA) cell line. The results showed the same conclusion as the bioinformatics analysis: BTN2/3 subfamilies were highly expressed in tumor tissues compared with normal brain tissue. The qRT-PCR results of cell lines showed that although the expression of BTN2/3 subfamilies in the two glioma cell lines was not completely consistent, they were generally higher than those in normal cell lines (**Figure 9B**).

To verify the immune infiltration of glioma tissues, we selected 4 GBM tissues for immunohistochemical fluorescence staining. The commercialized antibodies of BTN2/3 subfamilies only have BTN2A2 and BTN3A (non-specifically bind BTN3A1/2/3). We divided them into two groups (BTN3-high vs. low, BTN2A2-high vs. low) according to the qRT-PCR results of BTN2A2 and BTN3A1/2/3 of these 4 samples. CD68 (as a panmacrophage biomarker), BTN2A2, and BTN3 antibodies were applied to verify the immune infiltration of TAMs in GBM tissues. The VS120 virtual slide microscope system (Olympus, Tokyo, Japan) was utilized to control the uniform exposure time to quantify the fluorescence intensity (**Figure 9C**). The results showed that the green fluorescence (CD68) intensity representing TAMs was lower in tissues with low expression of BTN2A2 and BTN3A. However, in tissues with high expression of BTN2A2 and BTN3A, more significant immune infiltration of TAMs was observed.

## Discussion

It has been widely acknowledged that gliomas are typically accompanied by infiltrating lymphocytes and that their presence in the tumor site may correlate with an improved prognosis (12, 13). However, since naturally occurring T cells are observed less frequently in glioma (14), the pathophysiological characteristic of T-cell dysfunction in glioma makes it impractical to potentiate tumor regression on its own (15). Therefore, studying the unique immune mechanism mode of action in glioma may be the key to improving the current poor immunotherapy effect of glioma.

We have reported in a previous study that in LGG, FAM111A can be used as a new type of immune-related biomarker (5). When we reviewed the data, we found that the expression of the BTN/BTNL (butyrophilins/butyrophilin-like) family in LGG was highly associated with FAM111A, which attracted our attention. Recent findings have demonstrated the emergent role of butyrophilins/butyrophilin-like molecules (BTN/BTNL in humans and Btn/Btnl in mice) in the modulation of immune regulation (16), especially for the members of BTN3A, which are considered to dominate antitumor responses by coordinating alpha-beta and gamma-delta T cells (17). A recent study reported that BTN3A1-targeted therapy showed superior efficacy to PD-1 checkpoint therapy in validated orthotopic xenograft and syngeneic models of ovarian cancer (17). At the same time, in a variety of human cancers, BTN3A1 shows an excellent diagnostic value and can be used as a target for predicting drug resistance and judging the prognosis of patients (18–20). Nevertheless, whether the expression of the BTN/BTNL family or BTN2/3 subfamilies as a whole is associated with tumor immune infiltration in pan-glioma remains unknown.

The current study is the first to explore the expression and prognostic values of BTN2/3 subfamily members in pan-glioma. The results showed that five BTN2/3 subfamily members had higher expression levels in pan-glioma tumor tissue than in Genotype-Tissue Expression (GTEx) normal tissue. Furthermore, BTN2/3 subfamily expression was associated with the histological types of pan-glioma and correlated with tumor grade, 1p/19q codeletion, IDH status, and survival events. Further investigation is needed to confirm the role of BTN2/3 subfamily members as putative pan-glioma biomarkers. Analysis of data from GEPIA showed that the high expression levels of five BTN2/3 subfamilies were correlated with poor prognosis in pan-glioma, which was consistent with the hazard ratio (HR) survival map analysis result.

To further evaluate the function of the BTN2/3 subfamilies, we performed data analysis using GeneMANIA, CancerSEA, and STRING. CancerSEA analysis results showed that BTN2/3 subfamilies may influence the cell cycle, differentiation, and stemness of pan-glioma. GeneMANIA analysis results revealed that the antigen receptor-mediated signaling pathway response to IFN-γ and type I interferon were mainly enriched in BTN2/3 function enrichment. Previous studies have demonstrated that IFN-γ and TNF-α activate NF-κB, which in turn induces B7-H1 expression on myelodysplastic syndrome (MDS) blasts. At the same time, stemness is associated with a suppressed immune response (21), higher intratumoral heterogeneity, and dramatically worse outcomes for the majority of cancers (22). Glioma is characterized as a highly heterogeneous and poorly immunogenic cancer (23). Therefore, BTN2/3 subfamilies may promote pan-glioma development and progression through the IFN response and stemness regulation.

In recent years, tumor-associated immune cells have attracted increasing attention. Generally, tumor-associated immune cells can be classified into two types: tumor-antagonizing and tumor-promoting immune cells (24). Tumor-antagonizing immune cells consist of effector T cells, NK cells, DCs, M1-polarized macrophages, and N1-polarized neutrophils. Tumor-promoting immune cells include regulatory T cells and myeloid-derived suppressor cells (MDSCs). With respect to B cells, whose function is controversial in the tumor immune microenvironment, it is impracticable to simply summarize them into the binary category. In this study, BTN2/3 subfamily expression was positively associated with CD4+ T-cell, B-cell, neutrophil, macrophage, and DC infiltration levels. LGG (WHO grade II–III) is more correlated with immune infiltration than high-grade glioma (GBM; WHO grade IV). BTN2/3 subfamily expression may upregulate the infiltrating levels of CD4+ T and B cells to promote the development of pan-glioma.

ICIs have dramatically changed the landscape of therapies for cancers, especially in urologic cancer, melanoma, and gastrointestinal tumors, where promising therapeutic effects have been shown (25–27). ssGSEA analysis results showed that BTN2/3 subfamily expression was positively associated with CD274 (PD-L1), CD96, IDO1, IL10, IL10RB, PDCD1 (PD1), PDCD1LG2 (PD-L2), and TFGB1. TIMER immune infiltration analysis showed a significant correlation between BTN2/3 subfamily expression and most of the marker sets of Th1, Th2, Treg, and T-cell exhaustion. Currently, the blockade of PD-1 signaling and CTL4 signaling utilizing the PD-1/PD-L1 antibody and CTL4 antibody, respectively, has shown promising therapeutic effects in a variety of cancers, such as melanoma, non-small-cell lung cancer, renal cell cancer, and lymphoma (28–30). In this study, we found that the marker set of T-cell exhaustion was positively correlated apart from GZMB and HAVCR2 vs. BTN2A1. It is worth noting that PDCD1 was correlated with the BTN2/3 subfamilies in GBM but not statistically significant, and the correlation of PDCD1 in LGG was significantly stronger than that in GBM. A previous study reported that the glucocorticoid dexamethasone could upregulate CTLA4 mRNA and protein expression levels in CD4 and CD8 T cells and block CD28-mediated cell cycle entry and differentiation (30). In this study, we found that BTN2/3 subfamily expression was positively correlated with CTLA4 and PDL1. Therefore, BTN2/3 subfamily expression could possibly promote the development of pan-glioma by enhancing PD1 expression interacting with PDL1 and inhibiting CD4 and CD8 T-cell differentiation by upregulating CTLA4.

The most prevalent immune cells within brain tumors are macrophages (31), typically comprising up to ~30% of the tumor mass (32). In this study, the expression of the TAM-related marker CD68 was particularly related to the expression of the BTN2/3 subfamilies. TAMs within the brain tend to be protumorigenic and accumulate with higher tumor grades, and they are poor inducers of T-cell responses in glioma (32). The robust effects of TAM inhibition on blocking gliomagenesis have been reported (33). Activated TAMs have been shown to regulate GSC pools within the brain (34–36). These conclusions confirm our findings. The CD68 changes caused by the expression of BTN2/3 subfamilies may be involved in the regulation of stemness. The immunosuppressive effect induced by TAMs was also a pivotal factor for BTN2/3 subfamilies to promote the occurrence and development of glioma.

With the accumulation of clinical data and the popularization of sequencing technology, a large number of prognostic indicators, such as Pizeo1, GNG5, EVA1C, and lncRNA H19 (37–40), have shown excellent predictive performance. These indicators explain the occurrence and development mechanism of glioma from one or more perspectives and provide new evidence for the discovery and validation of new therapeutic targets. At the same time, the predictive and prognostic models of glioma patients based on clinicopathological characteristics and prognosis also provide an important reference for the treatment and management of the disease, making up for the limitations of high-throughput technology (41). Based on previous studies, in this study, we identified the potential functions and roles of the BTN2/3 subfamilies in the prognosis of glioma and as a novel biomarker of immune infiltration and explored the correlation with clinicopathological characteristics, explaining the mechanisms leading to panmacrophages participating in immune infiltration under the influence of BTN2/3 subfamilies.

In conclusion, increased BTN2/3 subfamily expression is correlated with a poor prognosis in glioma. In addition, it is positively associated with increased immune infiltration levels of CD4+ T cells, B cells, neutrophils, macrophages, and DCs. Our study provides promising therapeutic targets and novel biomarkers for pan-glioma.

## Materials and Methods

### Clinical Sample Preparation and High-Throughput Sequencing Analysis

A total of 144 GBM samples and 8 normal brain samples were collected from the Department of Neurosurgery of Shandong Provincial Hospital affiliated with Shandong University. The research was approved by the Research and Ethics Committee of Shandong Provincial Hospital. With the use of a high-throughput sequencing technique (Affymetrix GeneChip^®^ Human Transcriptome Array 2.0), clusters of differentially expressed PCGs in glioma specimens and paracancerous tissues were defined (**Table S1**). The suffix E-Signal represented the paracancerous tissues, and the suffix T-Signal represented the tumor tissues. Clusters in green indicate downregulated PCGs, and clusters in red indicate the opposite.

### Cell Culture and Reagents

The high-grade human glioma cell lines U251 and U87 and the normal human astrocyte cell line NHA were obtained from American Type Culture Collection (Manassas, VA, USA) and used for *in vitro* experiments. Tumor cells were maintained as monolayer cultures in Dulbecco’s modified Eagle’s medium (DMEM) supplemented with 10% fetal bovine serum (FBS), 100 units/ml of penicillin, and 100 μg/ml of streptomycin. The culturing environment was 37°C with 5% CO_2_.

### Quantitative Reverse-Transcription PCR

Briefly, total RNA was extracted from cell lysates or cytoplasm and nuclear extracts using TransZol Up (Transgen, Beijing, China). RNA was analyzed quantitatively using a NanoDrop (NanoDrop Technologies, Rockland, DE, USA). Total RNA (1 μg) was reverse transcribed into cDNA using a cDNA synthesis kit (Transgen, Beijing, China) according to the manufacturer’s instructions. RT-PCR was performed in a Bio-Rad CFX connect real-time system detector with Transgen SYBR Green Supermix. The reactions were analyzed using Bio-Rad CFX Maestro software (Version 4.1). The threshold cycles (CTs) were calculated, and the relative gene expression was analyzed after normalizing to GAPDH. Experimental-related primers were designed and produced by Takara (Maebashi, Japan). The primers were as follows:

BTN2A1
Forward primer (5′–3′): CTAGAAGGCCTCCTGTCCCT
Reverse primer (5′–3′): GTGTTTTCTCCAACCGTGGC
BTN2A2
Forward primer (5′–3′): GCGATTAATAGGAGGCCTCTGG
Reverse primer (5′–3′): AGATCCCATTCTCCTGGGCT
BTN3A1
Forward primer (5′–3′): TCTGGGGAGGGTGTATCCTG
Reverse primer (5′–3′): TCCTCCAGGAGCTTCACTCT
BTN3A2
Forward primer (5′–3′): ATACAGTGGAGCAACGCCAA
Reverse primer (5′–3′): GAAGGGGTCTGCGATGGAAA
BTN3A3
Forward primer (5′–3′): TCAGTGGGCTGTGATTTTCAGA
Reverse primer (5′–3′): TGGACCAAGAAGAGGGAGACA
GAPDH
Forward primer (5′–3′): TATGACAACAGCCTCAAGAT
Reverse primer (5′–3′): AGTCCTTCCACGATACCA

### Immunohistochemistry for Fluorescence Staining of Paraffin-Embedded Sections

The GBM tissues were washed with phosphate-buffered saline (PBS) buffer and fixed by immersion in a 10% formalin solution for 8 h at room temperature. Tissues were dehydrated in 70% (three times for 30 min)–90% (two times for 30 min)–100% (three times for 30 min) ethanol and finally immersed in xylene (mixed isomers) three times for 20 min each in room temperature. The tissue was embedded in paraffin at 58°C. Tissue sections (5 μm thick) were cut using a rotary microtome. The sections were floated in a 56°C water bath and mounted onto gelatin-coated histological slides. The slides were dried overnight at room temperature. The sections were rehydrated with xylene (mixed isomers) two times for 10 min each, and the slides were immersed in 100%–95%–70%–50% ethanol for 5 min. The slides were rinsed with double-distilled water (DDW) and rehydrated with PBS buffer for 10 min. The excess wash buffer was drained, and primary antibodies were applied as follows: anti-CD68 antibodies (mouse mAb, Abcam (Cambridge, UK; ab955), 1:50; rabbit pAb, Abcam (ab125212), 1:50), anti-BTN2A2 antibody (mouse mAb Abcam (ab233752), 1:100), anti-BTN3 antibody (rabbit pAb, ZEN-BIO (Sichuan, China; 251995), 1:50). Non-specific staining was blocked between the primary antibodies and the tissue by incubating the sample in blocking buffer (1% horse serum in PBS) for 30 min at room temperature. Primary antibodies were diluted in the incubation buffer according to the dilution mentioned above and incubated overnight at 4°C. After primary antibody incubation, the slides were washed 3 times for 15 min each in wash buffer. The slides were incubated with the secondary antibodies diluted in incubation buffer for 30 min at room temperature and then washed 3 times for 15 min each in wash buffer. Next, 300 μl of diluted DAPI solution was added to each slide and incubated for 5 min at room temperature. The slides were rinsed with PBS and mounted with antifade mounting media. Image scanning was performed using a VS120 virtual slide microscope (Olympus, Tokyo, Japan).

### BTN/BTNL and BTN2/3 Subfamily Expression Level Analysis

TCGA-GBMLGG was used and GTEx datasets were paired to investigate the expression of the BTN2/3 subfamily members across tumor and normal tissues. In addition, according to the expression status (basal expression level and the differential expression between tumor tissue and normal tissue), the BTN2/3 subfamily and its correlation in combination with clinicopathological characteristics were further analyzed by R software.

### BTN2/3 Subfamily Genomic Alterations and Correlation Analysis

An analysis of the cBio Cancer Genomics Portal (http://cbioportal.org) was performed, which is an open-access resource for the interactive exploration of multidimensional cancer genomic datasets (42, 43), to analyze BTN2/3 subfamily alterations in TCGA-GBMLGG sample. In addition, the portal was used to assess correlations among BTN2/3 subfamily members.

### Gene–Gene and Protein–Protein Interaction Networks

GeneMANIA (http://genemania.org/) is a fast gene network construction and function prediction tool for Cytoscape (44, 45), and STRING (https://string-preview.org/) is used for protein–protein interaction network functional enrichment analysis. Both tools were used to explore the BTN2/3 subfamily gene and protein network.

### Survival Analysis

GEPIA, an online database including TCGA gene expression data and clinical data (46, 47); LinkedOmics (http://www.linkedomics.org/login.php), a publicly available portal including multiomics data from 32 TCGA cancer types (48); and GEPIA were all used to examine correlations between BTN2/3 subfamily expression and OS/disease-free survival (DFS).

### Immune Inhibitor Analysis

TISIDB (http://cis.hku.hk/TISIDB/index.php), a web portal for tumor and immune system interactions that integrates multiple heterogeneous data types, was used to investigate correlations between BTN2/3 subfamily members and immune inhibitor.

### Tumor-Infiltrating Immune Cell Analysis

TIMER (https://cistrome.shinyapps.io/timer/), a web server for comprehensive analysis of tumor-infiltrating immune cells (49), was used to explore correlations between tumor-infiltrating immune cells and BTN2/3 subfamily expression.

### Statistical Analysis

Statistical analyses were conducted by using R software, and the results were analyzed by SPSS and GraphPad Prism 7.0. Univariate and multivariate analyses were used to assess the influence of clinical variables on survival. Two-tailed p-values less than 0.05 were considered statistically significant.

## Data Availability Statement

The original contributions presented in the study are included in the article/**Supplementary Material**. Further inquiries can be directed to the corresponding authors.

## Ethics Statement

The studies involving human participants were reviewed and approved by Research Ethics Committee of Shandong University. The patients/participants provided their written informed consent to participate in this study.

## Author Contributions

QL, QP, DH, and ZQ generated the hypothesis and designed the workflow. DH, ZQ, XJ, FY, YW, JG, and ZW performed the experiments. DH, ZQ, HF, and ZL interpreted the data. DH, ZL, and QL wrote the manuscript. QL and QP supervised the overall research, secured the funding, and interpreted the results. All authors listed have made a substantial, direct, and intellectual contribution to the work and approved it for publication.

## Funding

This research was supported by grants from the Natural Science Foundation of China, Grant No. 81771270 (QP) and Nos. 81871196 and 81471517 (QL); Shandong Provincial Natural Science Foundation, China, Grant No. ZR2020QH108 (FY); Key Technology Research and Development Program of Shandong, Grant No. 2019GSF107046 (QL); and Sciences and Technology Project of Jinan, Grant No. 202019088 (FY).

## Conflict of Interest

The authors declare that the research was conducted in the absence of any commercial or financial relationships that could be construed as a potential conflict of interest.

## Publisher’s Note

All claims expressed in this article are solely those of the authors and do not necessarily represent those of their affiliated organizations, or those of the publisher, the editors and the reviewers. Any product that may be evaluated in this article, or claim that may be made by its manufacturer, is not guaranteed or endorsed by the publisher.
