# Supplementary material for: Comprehensive Analysis of the Prognostic Value and Immune Infiltration of Butyrophilin Subfamily 2/3 (BTN2/3) Members in Pan-Glioma
Source: Front Oncol. 2022 Aug 10;12:816760. doi: 10.3389/fonc.2022.816760 (PMC9399357; doi:10.3389/fonc.2022.816760)
Supplement: Supplementary file 1 [file DataSheet_1.pdf]

Supplementary Figure and Figure caption

Figure S1

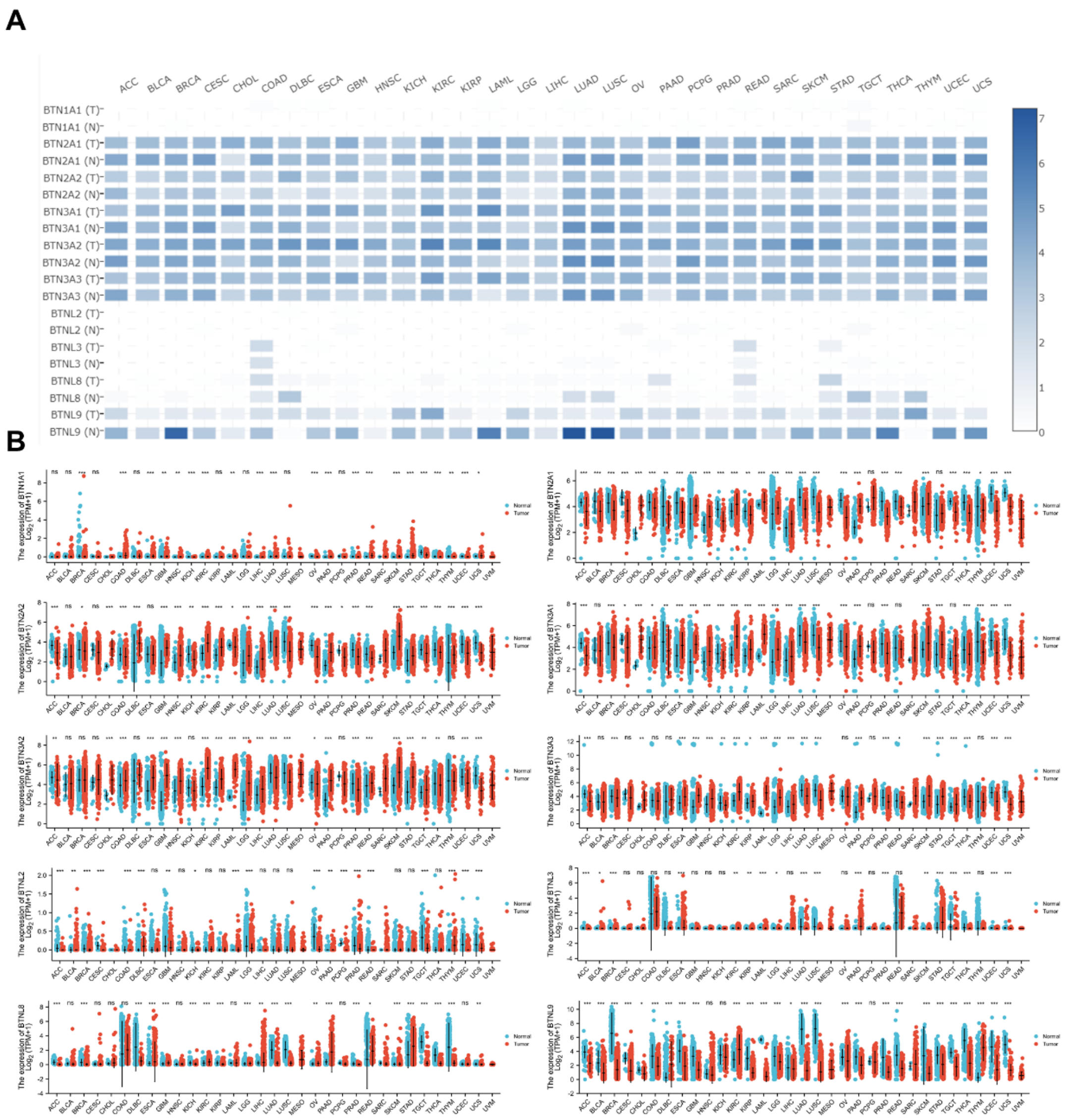

Figure S1 BTN/BTNL expressions profiles in pan-cancer

(A) The expression of BTN/BTNL in pan-cancer, tumors are compared with adjacent tumors or normal tissues.

(B) Scatter plot corresponding to Figure S1A
